# Fluorescently Tagged CCL19 and CCL21 to Monitor CCR7 and ACKR4 Functions

**DOI:** 10.3390/ijms19123876

**Published:** 2018-12-04

**Authors:** Vladimir Purvanov, Christoph Matti, Guerric P. B. Samson, Ilona Kindinger, Daniel F. Legler

**Affiliations:** 1 Biotechnology Institute Thurgau (BITg) at the University of Konstanz, CH-8280 Kreuzlingen, Switzerland; vladimir.purvanov@bitg.ch (V.P.); christoph.matti@bitg.ch (C.M.); guerric.samson@bitg.ch (G.P.B.S.); ilona.kindinger@bitg.ch (I.K.); 2 Faculty of Biology, University of Konstanz, D-78464 Konstanz, Germany

**Keywords:** fluorescent chemokines, chemokine receptors, CCL19, CCL21, CCR7, ACKR4, cell migration, leukocytes, cancer metastasis

## Abstract

Chemokines are essential guidance cues orchestrating cell migration in health and disease. Cognate chemokine receptors sense chemokine gradients over short distances to coordinate directional cell locomotion. The chemokines CCL19 and CCL21 are essential for recruiting CCR7-expressing dendritic cells bearing pathogen-derived antigens and lymphocytes to lymph nodes, where the two cell types meet to launch an adaptive immune response against the invading pathogen. CCR7-expressing cancer cells are also recruited by CCL19 and CCL21 to metastasize in lymphoid organs. In contrast, atypical chemokine receptors (ACKRs) do not transmit signals required for cell locomotion but scavenge chemokines. ACKR4 is crucial for internalizing and degrading CCL19 and CCL21 to establish local gradients, which are sensed by CCR7-expressing cells. Here, we describe the production of fluorescently tagged chemokines by fusing CCL19 and CCL21 to monomeric red fluorescent protein (mRFP). We show that purified CCL19-mRFP and CCL21-mRFP are versatile and powerful tools to study CCR7 and ACKR4 functions, such as receptor trafficking and chemokine scavenging, in a spatiotemporal fashion. We demonstrate that fluorescently tagged CCL19 and CCL21 permit the visualization and quantification of chemokine gradients in real time, while CCR7-expressing leukocytes and cancer cells sense the guidance cues and migrate along the chemokine gradients.

## 1. Introduction

Chemokines are best known for their central role in orchestrating cell migration. Chemokines and their cognate receptors not only regulate leukocyte trafficking during homeostasis and inflammation; they also guide and position cells in organogenesis and control the growth and spread of numerous tumors [1,2,3]. Chemokines constitute a family of about 50 small proteins of 8–12 kDa with only little sequence homology. However, they share a common three-dimensional structure termed the chemokine fold that is stabilized by two characteristic disulfide bridges between mainly four conserved cysteine residues [4]. Depending on the arrangement and spacing of the first two cysteines, they are divided into CC, CXC, CX_3_C, and XC chemokine subfamilies. Typical chemokine receptors are heptahelical class A G-protein coupled receptors (GPCRs) that signal through G_i_-proteins for guiding cell migration [5]. Ligand binding to chemokine receptors leads to the GDP/GTP exchange of coupled heterotrimeric G-proteins and the subsequent dissociation of the βγ-subunits from the α-subunit. The βγ-subunits then activate phospholipase C and phosphoinositide 3-kinase, leading to further activation of kinase cascades, including the extracellular signaling regulated kinases ERK-1/2, and to the generation of second messengers such as calcium from intracellular stores [5]. In addition, chemokine receptors can activate Src kinases and downstream signaling molecules such as the tyrosine phosphatase SHP2 [6]. The activity of chemokine receptors is commonly downregulated through phosphorylation of C-terminal serine and threonine residues, resulting in receptor desensitization, β-arrestin recruitment and receptor internalization [5].

The homeostatic CC chemokines CCL19 and CCL21 are constitutively expressed primarily by stroma cells in lymphoid organs [7,8,9]. In addition, CCL21 is produced by lymphatic endothelial cells. Its cognate receptor CCR7 is expressed on various subsets of immune cells including subpopulations of T cells and antigen-presenting dendritic cells (DCs). CCR7 and its ligands CCL19 and CCL21 play a crucial role in guiding antigen-bearing DCs and lymphocytes to lymphoid organs, thereby launching adaptive immune responses [8]. Misguidance of these leukocytes results in defective adaptive immune responses and can lead to autoimmune diseases, as shown in gene-targeted mice lacking CCR7 or its ligands [10]. Furthermore, if expressed by cancer cells, CCR7 facilitates cancer cell dissemination, migration and metastasis formation in lymphoid organs [11]. Interestingly, besides common signaling pathways of CCR7 by the two ligands, only CCL19 was shown to efficiently promote β-arrestin recruitment resulting in CCR7 internalization through clathrin-coated pits, whereas CCL21 is more proficient in inducing cell adhesion than CCL19 [9,12,13,14].

CCR7-guided cell migration is well studied for DCs. Upon pathogen encountering in peripheral tissues, they capture antigens and induce the expression of CCR7. DCs then migrate in a CCR7-dependent and haptotactic manner along an immobilized CCL21 gradient towards lymphatic vessels from where they are flushed into the sinus of the draining lymph node [15,16,17]. Although information on how a chemokine gradient is formed in vivo is scarce, it is well accepted that CCL21 binds to glycosaminoglycans (GAGs). For CCL21, this occurs through its unique extended C-terminal tail that includes stretches of basic amino acids and is therefore negatively charged [18,19,20]. The interaction of CCL21 with GAGs on lymphatic endothelium is critical for restricting the chemokine distribution and thus contributes to shape immobilized chemokine gradients [15]. Notably, CCL19 lacks high affinity binding to GAGs and hence is considered to diffuse or form soluble gradients. In vitro, it has been shown that the migration of DCs on homogenously immobilized CCL21 can be directed by applying soluble CCL19 locally [13]. Exploiting different microfluidic devices revealed that DCs differentially respond to competing chemokine gradients and that cell guidance varies depending on whether chemokine gradients are soluble or immobilized [21,22].

The generation and maintenance of local chemokine gradients is critical for efficient leukocyte trafficking. A family of atypical chemokine receptors (ACKRs) was recently identified to internalize and degrade chemokines, thereby ACKRs regulate the local availabilities of chemokines and shape chemokine gradients [23,24]. ACKRs share ligands and structure of typical chemokine receptors, but do not couple to G_i_-proteins for signal transduction and hence fail to induce cell migration. However, ACKRs scavenge chemokines and deliver them for lysosomal degradation [25]. ACKR4 (formerly also known as CCRL1 or CCX-CXR) is expressed by lymphatic endothelial cells lining the ceiling of the subcapsular sinus of lymph nodes and scavenges the CCR7 ligands CCL19 and CCL21, as well as CCL25 [26]. In fact, ACKR4 has been shown to create CCL21 gradients across the sinus floor of lymph nodes and thereby enables directional migration of DCs [26].

So far, quantification of chemokine gradients was very challenging and had profound limitations: (i) quantification was often restricted to fixed specimen for staining purposes of the chemokines using antibodies, (ii) chemokines had to be printed as gradient to a substrate using highly sophisticated methods, or (iii) chemokine gradients were simply estimated by concurrently applying dyes, e.g., FITC-dextran, with the chemokine. Moreover, the scavenging activity of chemokine receptors was routinely determined by measuring liberated ^125^I from iodinated chemokines upon receptor-mediated internalization and lysosomal degradation [27]. Here, we designed and generated functional fluorescently labeled chemokines by fusing CCL19 and CCL21 to monomeric red fluorescent protein (mRFP), revealing CCL19-mRFP and CCL21-mRFP, as powerful tools to visualize and characterize CCR7 and ACKR4 functions in a spatiotemporal manner using time-lapse video microscopy.

## 2. Results

### 2.1. Design of Fluorescently Tagged CCL19 and CCL21

**Figure 1:**
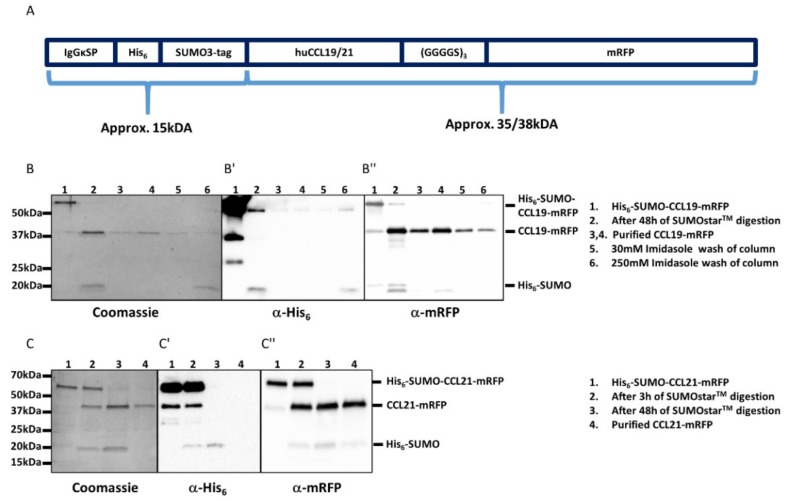
Design and purification of CCL19-mRFP and CCL21-mRFP. (**A**) Graphic scheme of the constructs used to generate fluorescently tagged full-length chemokines. (**B**,**C**) Coomassie-stained SDS-PAGE gels (left) and corresponding Western blots (middle and right) of samples collected during the purification steps of CCL19-mRFP (**B**) and CCL21-mRFP (**C**). Secreted His_6_-SUMO-CCL19-mRFP was collected from the supernatants of transfected HEK293 cells and affinity purified over a Ni^2+^ column (**B**, line 1). The His_6_-SUMO tag was cleaved off by incubation for 48 h with SUMOstar™ protease at 8 °C (**B**, lane 2) and applied again on the Ni^2+^ column to remove the His_6_-SUMO tag. The purified CCL19-mRFP was collected as flow-through (**B**, lanes 3,4). The column was washed with 30mM imidazole (**B**, lane 5) and 250 mM imidazole to completely remove the His_6_-SUMO tag from the column for reuse (**B**, lane 6). Similarly, secreted His_6_-SUMO-CCL21-mRFP (**C**, lane 1), was digested with SUMOstar™ protease for 3 and 48 h (**C**, lanes 2,3) and applied to a Ni^2+^ column. Purified CCL21-mRFP was collected as flow-through (**C**, line 4). Western blots of the same samples using anti-His tag (**B′**,**C′**) or anti mRFP (**B″**,**C″**) antibodies are shown.

The availability of fluorescently labeled chemokines is highly desired but very limited. Chemokines can be expressed and produced in inclusion bodies of bacteria as recombinant proteins, but the denatured purified chemokine must be properly refolded and contaminating LPS derived from host cells needs to be carefully removed [4,27,28]. Moreover, the N-terminus of recombinant chemokines must be processed and freely available to render the chemokine functional. We decided to elaborate a strategy to easily and economically produce native fluorescently tagged chemokines in a mammalian cell expression system, such as HEK293 cells. To achieve this we designed an expression system in which we fused the cDNA coding for the mature, full-length forms of human CCL19 (amino acids 22–98) and CCL21 (amino acids 24–134) at their C-terminus to a flexible short linker sequence (coding three times for the amino acids: GGGGS, single letter code) followed by mRFP (Figure 1A). In addition, fusing an N-terminal signal peptide (derived from mouse IgGκ) followed by a His_6_-SUMO double tag to the mature form of the chemokines entails three advantages: (i) the chemokine fusion protein is secreted as properly folded protein upon overexpression in mammalian cells. (ii) the His_6_ tag enables easy affinity purification of the fusion protein over a Ni^2+^-column. (iii) the SUMO3 tag renders the recombinant protein soluble and can be simply and precisely cleaved off with an impeccable specificity and efficiency using the SUMO protease 2 (SUMOstar™), liberating the native chemokine-mRFP with its correct mature N-terminus [4,28].

### 2.2. Purification and Functional Characterization of CCL19-mRFP and CCL21-mRFP

We transiently transfected HEK293 cells with either pHis_6_-SUMO-CCL19-mRFP or pHis_6_-SUMO-CCL21-mRFP and collected the supernatants after three and five days of culture, as described in Section 4. The secreted chemokine fusion proteins were purified in a single step over a Ni^2+^-column. The His_6_-SUMO tag was cleaved off the chemokine and removed by a subsequent affinity purification step using the same Ni^2+^-column as before, yielding about 25‒50 µg pure, native and mature human CCL19-mRFP or CCL21-mRFP per T175 cell culture flask (Figure 1B,C).

**Figure 2:**
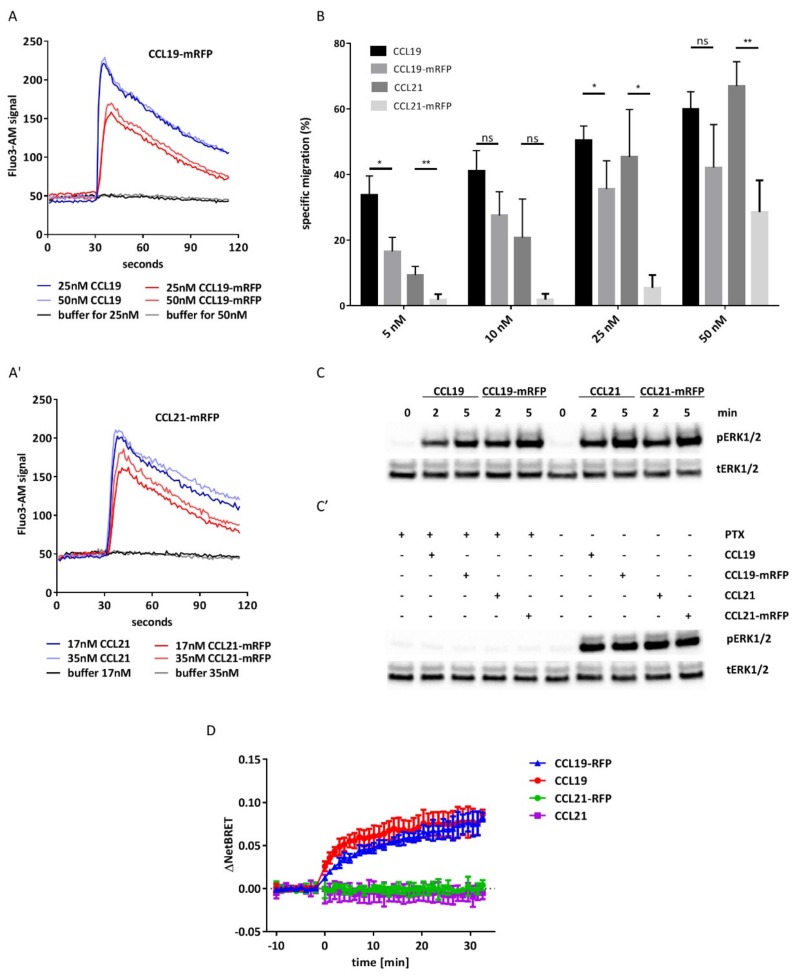
CCL19-mRFP and CCL21-mRFP are functional chemokines. (**A**) Real-time changes in [Ca^2+^]_i_ concentrations in CCR7 transfected 300-19 cells in response to graded concentrations of CCL19 and CCL19-mRFP (A) and CCL21 and CCL21-mRFP (**A′**). A representative experiment out of three is shown. (**B**) Transwell chemotaxis of human mature MoDCs in response to chemokines. Human mature MoDCs were allowed to migrate in response to gradient concentrations of chemokines for 180 min. Migrated cells were counted and percentages of specifically migrated cells relative to the input were calculated. Mean values and SEM of duplicates derived from three independent experiments are shown. (**C**) Stimulation of human mature MoDCs with CCL19-mRFP and CCL21-mRFP results in the phosphorylation of ERK1/2. Human mature MoDCs were stimulated with 50 nM of CCL19, CCL19-mRFP or CCL21-mRFP for the indicated time points. ns: statistically not significant; * *p* < 0.05; ** *p* < 0.005. (**C’**) MoDCs were pre-treated with 200 ng/mL PTx for 3 h and subsequently stimulated with 50 nM of chemokines for 5 min. Phosphorylation of ERK1/2 (pERK1/2) was determined by Western blotting. Re-probing the blots for total ERK1/2 (tERK1/2) served as a control for equal protein loading. (**D**) Chemokine-induced β-arrestin recruitment to CCR7. β-arrestin recruitment was determined by BRET upon stimulation with 50 nM of indicated chemokine. Mean values ±S.D. of 2 (CCL21, CCL21-mRFP) or 3 (CCL19, CCL19-mRFP) independent experiments with technical duplicates are shown.

We tested the activity and functionality of the purified CCL19-mRFP and CCL21-mRFP by first assessing chemokine-mediated calcium mobilization. To this end, we used 300-19 pre-B cells stably expressing human CCR7 [19,29,30,31]. Stimulation of CCR7-expressing cells with CCL19-mRFP or CCL21-mRFP, as well as with CCL19 or CCL21, elicited transient elevations of [Ca^2+^]_i_ typical for ligand-induced chemokine receptor activation (Figure 2A). No chemokine-mediated calcium mobilization was observed in parental 300-19 cells that do not express CCR7 (not shown). Next we tested the fluorescently tagged chemokines for their ability to induce migration of human mature monocyte-derived DCs (MoDCs) using classical 2D-Transwell chemotaxis assays. Both fluorescently tagged chemokines recruited human mature MoDCs, although with a slightly reduced efficacy as compared to non-tagged versions of the chemokines (Figure 2B). Notably, at physiological concentrations of 50 nM, both CCL19-mRFP and CCL21-mRFP specifically attracted human mature MoDCs. In addition, stimulation of human MoDCs with 50 nM of fluorescently tagged and untagged chemokines resulted in the activation of ERK-1/2 (Figure 2C). Pre-treating human MoDCs with pertussis toxin (PTx) to inhibit G_i_ protein-coupling to CCR7 abolished chemokine-mediated ERK1/2 phosphorylation (Figure 2C). Consistent with ligand-biased CCR7 signaling [9,12,13], only CCL19 and CCL19-mRFP (at 50 nM each) efficiently recruited β-arrestin to CCR7 (Figure 2D).

These data clearly indicate that CCL19-mRFP and CCL21-mRFP are functional fluorescent chemokines with preserved biased signaling capabilities.

### 2.3. CCL19-mRFP Is Internalized by CCR7 and ACKR4, whereas CCL21-mRFP Is Preferentially Scavenged by ACKR4

**Figure 3:**
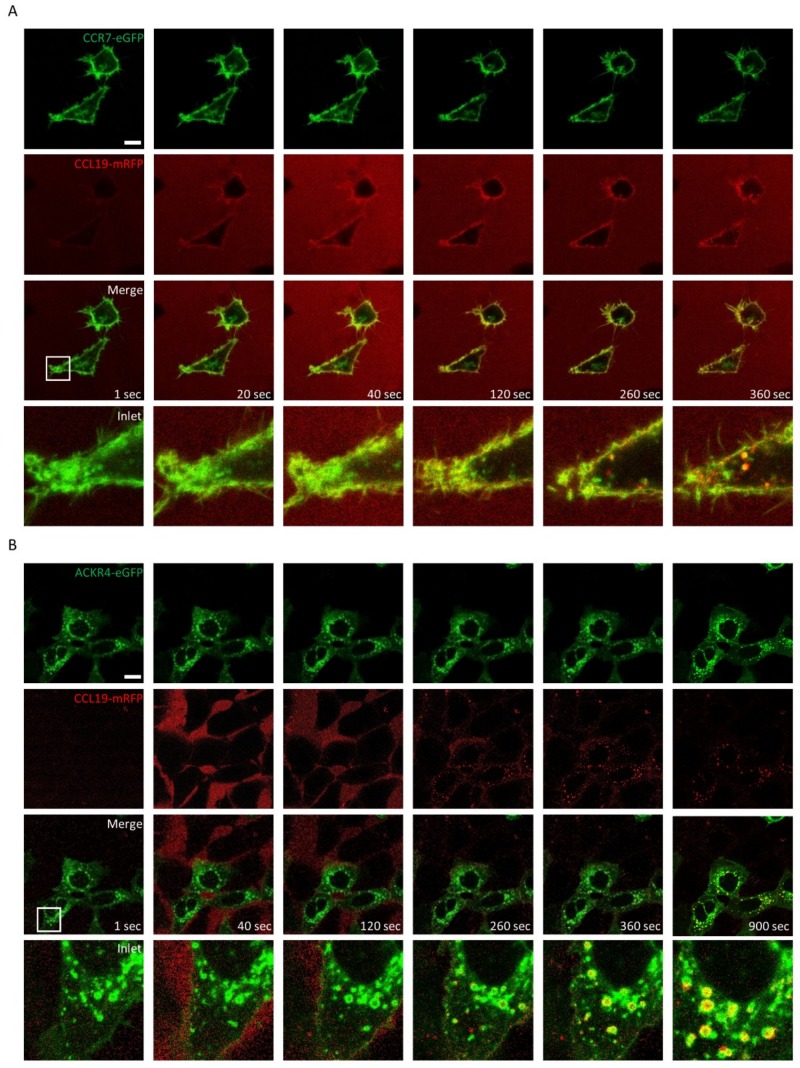
Internalization of CCL19-mRFP by CCR7-eGFP and ACKR4-eGFP. 100 nM of CCL19-mRFP was added to HEK293 cells transiently transfected with CCR7-eGFP (**A**) or ACKR4-eGFP (**B**) and CCL19-mRFP internalization was recorded by time-lapse video microscopy. Series of images captured at indicated time points are illustrated (scale bar: 10 µm). Frames: magnification of images.

In order to exploit our fluorescently tagged chemokines to study endocytosis, we performed time-lapse video microscopy using HEK293 cells as a model system that transiently express either CCR7-eGFP or ACKR4-eGFP. Applying CCL19-mRFP to the medium of transiently transfected HEK293 cells resulted in chemokine binding to cells expressing CCR7-eGFP, but not to untransfected cells. CCL19-mRFP subsequently, within seconds, was internalized together with the receptor and accumulated within a few minutes in vesicular structures where the chemokine and the receptor co-localized (Figure 3A).

ACKR4 is known to steadily cycle between the plasma membrane and endomembrane compartments even in the absence of ligands. Addition of CCL19-mRFP to HEK293 cells transiently transfected with ACKR4-eGFP was readily internalized exclusively by those cells expressing the atypical chemokine receptor (Figure 3B and Supplementary Video 1). Similar results were obtained in HeLa cells expressing high amounts of ACKR4-eGFP, which consequently scavenged CCL19-mRFP in an impeccably efficient manner (Supplementary Video 2).

**Figure 4:**
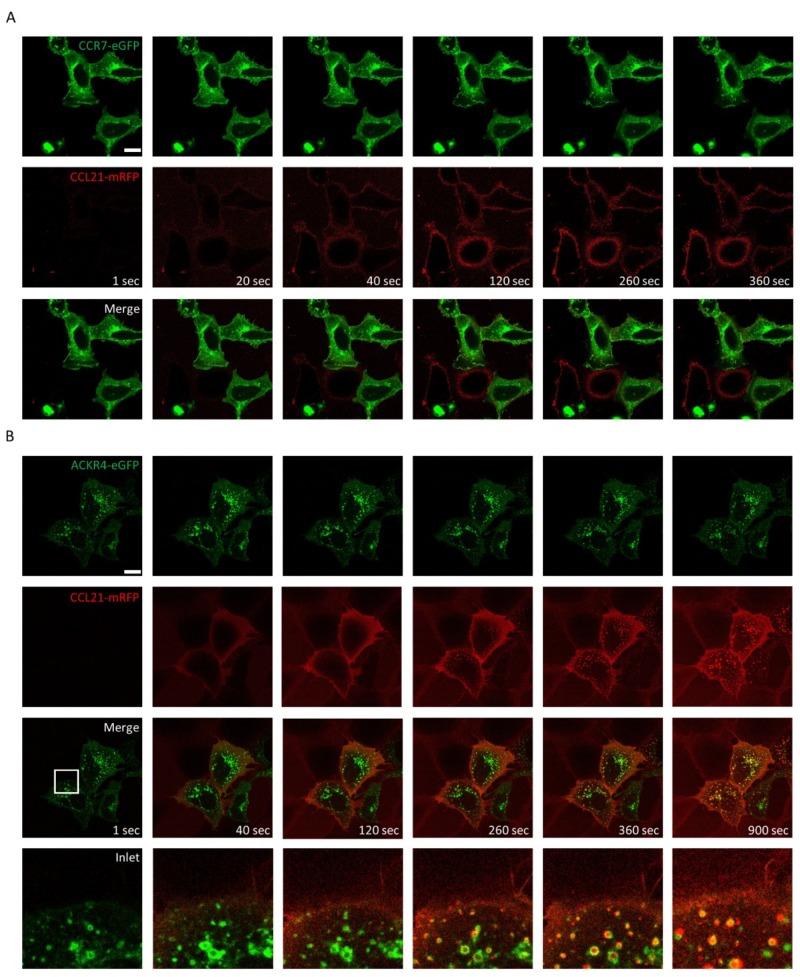
Monitoring CCL21-mRFP binding to CCR7-eGFP and internalization by ACKR4-eGFP. 100nM of CCL21-mRFP was added to HEK293 cells transiently transfected with CCR7-eGFP (**A**) or ACKR4-eGFP (**B**) and CCL21-mRFP binding and receptor trafficking was recorded by time-lapse video microscopy. Series of images captured at indicated time points are depicted (scale bar: 10 µm). White frames: magnification of images.

Addition of CCL21-mRFP to HEK293 cells expressing CCR7-eGFP bound to surface CCR7, but was barely internalized (Figure 4A), as expected and previously described for untagged CCL21 [12]. In contrast, CCL21-mRFP was rapidly internalized by HEK293 cells expressing ACKR4-eGFP (Figure 4B). These data illustrate that CCL19-mRFP and CCL21-mRFP are versatile tools to spatiotemporally study chemokine internalization and receptor trafficking.

### 2.4. Monitoring DC Migration in 3D Collagen along Gradients of CCL19-mRFP or CCL21-mRFP by Time-Lapse Video Microscopy

**Figure 5:**
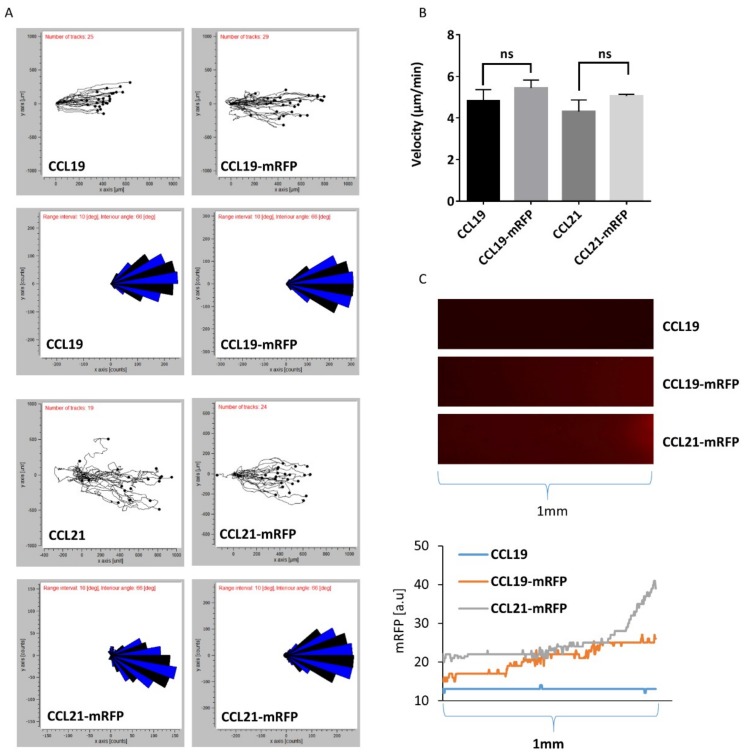
DC migration in 3D collagen along gradients of CCL19-mRFP and CCL21-mRFP. Mouse mature bone-marrow-derived DCs were imbedded in 3D collagen in µ-slide chemotaxis chambers. CCL19, CCL19-mRFP, CCL21, or CCL21-mRFP at 100 nM concentration was applied to the right reservoir. (**A**) Combined rose diagrams and tracks of single cells migrating towards higher concentrations of chemokines from a representative experiment are shown. (**B**) Mean velocity of individually migrating cells derived from three independent experiments were quantified. ns: statistically not significant. (**C**) Gradients of CCL19-mRFP and CCL21-mRFP from (**A**) were visualized by fluorescence microscopy and enumerated by determining the fluorescent intensity derived from the corresponding mRFP signal.

Next, we addressed whether we can monitor DC migration through a 3D collagen matrix along gradients of fluorescently tagged chemokines by time-lapse video microscopy. To achieve this, we used commercially available µ-slides chemotaxis chambers (from Ibidi) where mouse mature bone-marrow-derived DCs are imbedded into a 3D collagen matrix within a narrow observation area connected to two larger reservoirs to one of which we applied 100 nM of chemokine to establish a gradient that is stable for at least 48 h based on the manufacturer’s information. Migrating DCs were recorded by time-lapse video microscopy, tracked and analyzed using the ‘chemotaxis and migration tool’ provided by the manufacturer. Figure 5A depicts directional migration patterns of DCs as spider and rose diagrams. DC migration through 3D collagen along gradients of CCL19, CCL19-mRFP, CCL21, or CCL21-mRFP was comparable as equal velocity was recorded (Figure 5B). Simultaneously, gradients of the fluorescently tagged chemokines were visualized and the intensity of the mRFP signal was enumerated (Figure 5C). These data suggest that although the same concentration of chemokine was applied in the reservoir, CCL21-mRFP formed steeper gradients than CCL19-mRFP, at least near the reservoir, which can be explained by the stickiness of CCL21 and its local immobilization.

### 2.5. Monitoring CCR7-Dependent Cancer Cell Migration in 3D Collagen along a CCL19-mRFP Gradient

**Figure 6:**
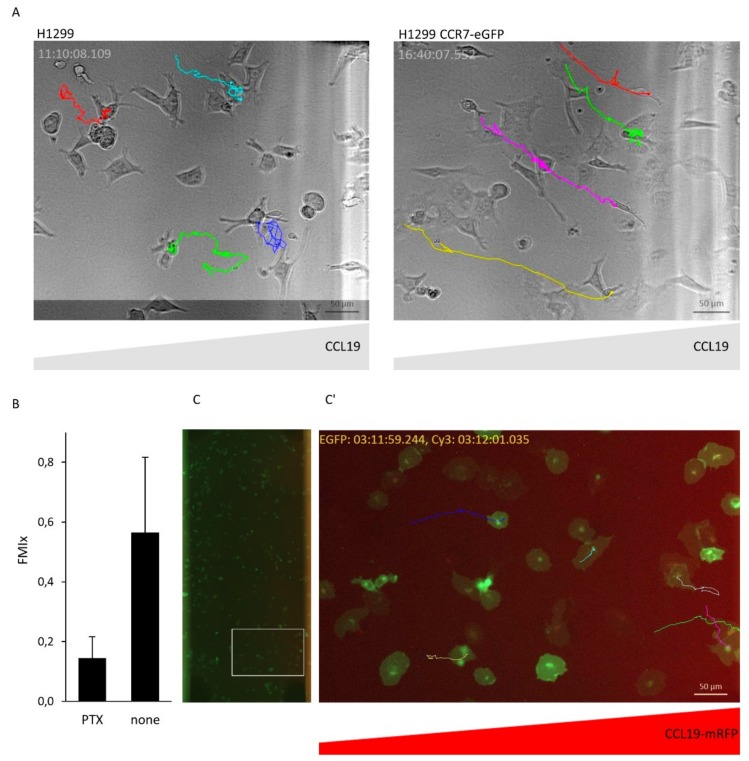
CCR7-dependet migration of H1299 cancer cells along CCL19 and CCL19-mRFP gradients. (**A**) H1299 wild-type cells and H1299 cells expressing CCR7-eGFP were imbedded in 3D collagen in µ-slide chemotaxis chambers. CCL19 (100 nM) was added to the right reservoir and cell migration monitored by time-lapse video microscopy. Colored tracks of individual cells are depicted. (**B**) H1299 cells expressing CCR7-eGFP were untreated or treated with PTx to inhibit G_i_-dependent chemokine signaling and subjected to cell migration assays as described in (**A**). Mean forward migration index (FMIx) and S.D. of individually migrating cells derived from two independent experiments are indicated. (**C**) H1299 cells expressing CCR7-eGFP were imbedded in 3D collagen in µ-slide chemotaxis chambers, CCL19-mRFP was added to the right reservoir. Cancer cell migration was monitored by time-lapse video microscopy by depicting tracks of individual cells. CCL19-mRFP gradient was visualized simultaneously by its fluorescence (scale bar: 10 µm).

As cancer cells can utilize CCR7 to metastasize in lymph nodes [11], we used the human non-small cell lung carcinoma cell line H1299 expressing CCR7-eGFP or not as model to study cancer cell migration. H1299 cells imbedded in 3D collagen migrated only along CCL19 gradients if they express CCR7 (Figure 6A and Supplementary Videos 3 and 4). Preventing G_i_-protein signaling by incubating H1299 cells expressing CCR7-eGFP with PTx abolished directional migration towards CCL19 (Figure 6B). Finally, we exposed H1299 cells expressing CCR7-eGFP imbedded in 3D collagen to a gradient of CCL19-mRFP. CCR7-eGFP expressing H1299 cells persistently migrated along the CCL19-mRFP gradient (Figure 6C and Supplementary Video 5).

Taken together, we demonstrated in this study that CCL19 and CCL21 can be produced as functional fluorescently tagged proteins in mammalian cells. Purified CCL19-mRFP and CCL21-mRFP therefore represent versatile tools to study chemokine and receptor functions, such as receptor trafficking and chemokine scavenging, in real-time using time-lapse video microscopy. Moreover, the fluorescent tags fused to CCL19 and CCL21 permits visualization of chemokine gradients in vitro without perturbing the chemokine functions, while cells are sensing them and migrate along the gradients.

## 3. Discussion

In the present study, we demonstrated that the homing chemokines CCL19 and CCL21 can be functionally expressed in and secreted from mammalian cells as fluorescently tagged fusion proteins. We provided experimental evidence that CCL19-mRFP and CCL21-mRFP induce typical chemokine receptor signaling pathways such as mobilization of calcium or activation of the ERK-1/2 pathway in cell lines, as well as in primary cells. Moreover, the fluorescently tagged chemokines were perfectly suitable to monitor chemokine internalization by CCR7 and ACKR4, as well as receptor trafficking in real-time as assessed by time-lapse video microscopy. Furthermore, measuring the fluorescent intensity signal derived from the mRFP directly fused to the chemokine allowed us to visualize and quantify the CCL19 and CCL21 gradients in real time while cells were exploring the presented guidance cue. We observed that under the same experimental condition the CCL21-mRFP gradient is steeper than the CCL19-mRFP one, at least close to the reservoir releasing the chemokine. Such exponential CCL21 gradients over about 90 µm were also found in vivo, namely in the mouse skin where the CCL21 gradient was establish between the perilymphatic interstitium towards the chemokine-producing lymphatic endothelial cells forming lymph vessels [15]. Notably CCL19-mRFP gradients seem to be rather linear reflecting the soluble biochemical properties of CCL19. This observation is in line with the diffusion based 10 kDa-FITC-dextran gradient [22] or the rhodamine and fluorescein based gradients [21] that were used to predict soluble CCL19 gradients in previous studies. Our data suggest that estimating chemokine gradients in in vitro assays using FITC-dextran or other soluble molecules and/or dyes may only give limited and non-precise information on the nature of the real chemokine gradient. The use of fluorescently tagged chemokines, such as CCL19-mRFP or CCL21-mRFP, will now allow accurate measurements and quantification of chemokine gradients under various experimental conditions.

Furthermore, these fluorescently tagged chemokine can be used to study the endocytosis routes taken by classical and atypical chemokine receptors. CCL19 is known to be internalized together with CCR7 via clathrin-coated pits [12]. It is also known that internalized CCR7 recycle back to the plasma membrane, whereas co-internalized CCL19 becomes sorted to lysosomal degradation [12]. How and where the chemokine and its receptor are separated to be differentially sorted remains to be investigated. Remarkable, CCL21 is barely internalized by CCR7. Both ligands have similar binding affinities to CCR7 [31], but induce common as well as biased signaling [9]. The molecular details how this works are still largely unknown. The fluorescently tagged chemokine may permit to address the unanswered fundamental questions.

Interestingly, migrating monocytes were shown to scavenge CCL2-mCherry released from a microinjection needle [32]. Whether CCL19 is also internalized by migrating cells remains to be addressed. The fluorescently tagged CCL19 and CCL21 will allow future investigation to address the question whether such potential chemokine scavenging during migration modulates gradients and whether scavenging contributes to directional migration. These new tools will in addition permit to investigate potential differences in amoeboid single cell modes of migration used by leukocytes and mesenchymal-type collective cell migration of cancer cells. In vivo, cells are exposed to different chemokines and it remains largely unknown how cells navigate in the presence of different guidance cues. In vitro systems, including microfluidic devices, in combination with differentially fluorescently labeled chemokines will permit to address such fundamental cell biological concepts in future.

## 4. Materials and Methods

### 4.1. Reagents and Antibodies

The following reagents were purchased as indicated: recombinant human CCL19 and CCL21 (PeproTech, Rocky Hill, CT, USA), monoclonal anti-polyhistidine antibodies conjugated to HRP (Sigma, A7058-1VL, St. Louis, MO, USA), rabbit-anti-mRFP (PM005, MBL International, Woburn, MA, USA), anti-phospho-p44/42 MAPK (ERK1/2; pThr202/Tyr204; Cell Signaling Technology #4370, Danvers, MA, USA), anti-p44/42 MAPK (ERK1/2) Antibody (Cell Signaling Technology #9102), peroxidase conjugated AffiniPure goat-anti-rabbit IgG (Jackson Immunoreseach, West Grove, PA, USA), Fluo-3 AM (Molecular Probes, Eugene, OR, USA), Pertussis Toxin (Tocris, Bristol, UK).

### 4.2. Cloning, Expression, and Purification of CCL19-mRFP and CCL21-mRFP

The signal peptide of IgGκ (coding for the amino acids: METDTLLLWVLLLWVPGSTG) was ligated to the N terminus of the cDNA encoding His_6_-SUMO3 followed by the human mature codon optimized cDNA of CCL19 (amino acids 22‒98) and sub-cloned in pcDNA3-mRFP with a (GGGGS)_3_ flexible linker revealing pHis_6_-SUMO-CCL19-mRFP plasmid. To clone pHis_6_-SUMO-CCL21-mRFP, a second BamHI restriction site was introduced by site directed mutagenesis into pHis_6_-SUMO-CCL19-mRFP using the primers 5′-GCAGATCGGCGGATCCACCAACGACGCCGAGGATTG and 5′-CAATCCTCGGCGTCGTTGGTGGATCCGCCGATCTGC. The cDNA fragment of CCL19 was replaced by a PCR fragment coding for human mature CCL21 (amino acids 24–134) using the primers 5′-GACCCAAGGATCCGATGGAGGGGCTCAGGACTG and 5′-GTCGACGGATCCGAATTCTGGCCCTTTAGGGGTCTG.

HEK293 cells were grown in DMEM medium supplemented with 10% FCS (Lonza) and 1% penicillin/streptomycin (Biowest, San Marcos, TX, USA) in T175 flasks. Cells were transfected with either pHis_6_-SUMO-CCL19-mRFP or pHis_6_-SUMO-CCL21-mRFP using FuGENE^®^ 6 transfection reagent (Promega, Madison, WI, USA). The next day medium was changed to DMEM supplemented with only 5% FCS and cultured for another three days. The supernatants containing the secreted chemokines were collected and cells were grown for an additional two days in fresh medium before harvesting it again. Pooled supernatants were centrifuged to remove cell debris and supplemented with 20 mM imidazole before applying on 1 mL Ni^2+^ agarose matrix packed in a gravity column previously equilibrated with binding buffer (20 mM Tris-HCl pH 7.5, 500 mM NaCl, and 20 mM imidazole). Ni^2+^ columns were washed with 10 column volumes of washing buffer (20 mM Tris-HCl pH 7.5, 500 mM NaCl, and 30 mM imidazole) and the total captured protein was collected by two sequential elutions with an elution buffer (20 mM Tris-HCl pH 7.5, 500 mM NaCl, and 250 mM imidazole). The column was re-equilibrated with washing buffer for the second purification step. The eluted His_6_-SUMO-chemokine-mRFP proteins were applied on Vivaspin 20 cross-flow spin columns (Sartorius, Göttingen, Germany) with a cutoff of 10 kDa for chemokine concentration and to exchange for the washing buffer. His_6_-SUMO-chemokine-mRFP proteins were digested using SUMOstar™ protease (LifeSensors, Malvern, PA, USA) for up to 48 h at 8 °C. The digested material was applied onto the re-equilibrated Ni^2+^ column to retain the His_6_-SUMO tag and the impurities of the first purification step. The flow-through containing the pure CCL19-mRFP or CCL21-mRFP, respectively, was collected. The chemokine-mRFP containing fractions were concentrated and the buffer was exchanged to PBS on Vivaspin 20 cross-flow spin columns. The last flow-through fraction was collected as negative control buffer solution.

### 4.3. Cloning and Expression of Chemokine Receptors

Human ACKR4 cDNA was amplified by PCR using the primers 5′-GAACAAGCTTCATTACGGCCGCTTTGGA and 5′-CTACCTCGAGCCCCAATAGAGAAGGTAGAAGT using a synthetic cDNA (ID: 11AA4G6P; Life Technologies) as template. The PCR product was inserted into the HindIII and XhoI restriction sites of pcDNA3. Site-directed mutagenesis was performed to re-introduce a methionine at position 1 using the primers 5′-CCCAAGCTTCATTACGATGGCTTTGGAACAAAATC and 5′-GATTTTGTTCCAAAGCCATCGTAATGAAGCTTGGGTC. Next, ACKR4 was amplified using 5′-GGAGACCCAAGCTTCATTACGATGGC (Primer-A) and 5′-GAGCTCGAGTCCACCAATAGAGAAGGTAGAAGTAGGTTCAGTTGGACC. In addition, eGFP was amplified from CCR7-eGFP [12] and a GGLES(GGGGS)_3_ linker introduced using the primers 5′-GGACTCGAGAGCGGAGGTGGCGGTTCTGGTGGTGGCGGTTCCGGCGGTGGCGGTAGCGTGAGCAAGGGCGAGGAGCTG and 5′-GCCCTCTAGACTACTTGTACAGCTCGTCC (Primer-B). Both PCR products were digested with XhoI, ligated and PCR amplified with Primer-A and Primer-B, digested with HindIII and XbaI, and inserted into pcDNA3.

CCR7-eGPF and ACKR4-eGFP were transiently expressed in HEK293, HeLa, or stably expressed in H1299 cells. HEK293 and H1299 cells were grown in a DMEM medium supplemented with 10% FCS and 1% penicillin/streptomycin, whereas HeLa cells were cultured in RPMI1640 medium with 10% FCS and 1% penicillin/streptomycin.

### 4.4. Preparation of Monocyte-Derived and Bone-Marrow-Derived DCs

Human monocyte-derived DCs were generated and matured with a cocktail of inflammatory cytokines for 48 h as described previously [6]. Mouse-bone-marrow-derived DCs were generated and matured with LPS for 24 h as described before [33].

### 4.5. Chemotaxis Assays

Two-dimensional chemotaxis assays were performed in duplicate in 24-well Transwell™ chemotaxis chambers (Costar^®^ Permeable support, 3421, Washington, DC, USA) with 5 μm pore-sized polycarbonate membranes. Mature MoDC from different donors (10^5^ per well) in medium were allowed to migrate to the lower well containing graded concentrations of chemokines for 180 min at 37 °C in a 5% CO_2_ atmosphere. Migrated cells were harvested and enumerated by flow cytometry (LSRII, BD Biosciences, San Jose, CA, USA). The number of cells that migrated spontaneously to the lower compartment in the absence of chemokines was subtracted and the percentage of migrated cells was calculated relative to the input of cells.

Three-dimensional migration through collagen was performed in µ-slide chemotaxis chambers purchased from Ibidi (Martinsried, Germany) according to the manufacturer’s protocol. Briefly, mature bone-marrow-derived DCs or H1299 cells were collected and re-suspended in RPMI1640 or DMEM supplemented with 10% FCS and 1% penicillin/streptomycin, respectively, to 10^7^ cells/mL. Twenty microliters 10× DMEM, 10 μL 7.5% NaHCO_3_, 2 μL 10N NaOH, and 150 μL PureCol collagen I (Advanced Biomatrix) were premixed, added to 90 μL cell suspension, and mixed carefully. The mixture was applied to µ-slide chemotaxis chambers and mounted as described in the manufacturer’s instructions. Chemokines were applied to the right reservoir, whereas control buffer from the chemokine purification was added to the left reservoir. Cell migration was monitored by time-lapse video microscopy on a Zeiss Axiovert 200M (Oberkochen, Germany) equipped with a Tokai Hit INU (Shizuoka Japan) incubation system for at least 3 h for DCs or overnight for H1299 cells. Cell migration was analyzed and quantified using the ‘chemotaxis and migration tool’ software provided by Ibidi. At least 24 cells per condition for every experiment were analyzed. The fluorescent mRFP gradient was stable and recorded before and after the migration assay.

### 4.6. Calcium Flux

Changes in concentrations of intracellular free calcium was determined in 300-19 pre-B cells expressing CCR7 loaded with Fluo-3-AM and recorded as previously described [12]. As a negative control, a solution from the last chemokine purification step was used.

### 4.7. β-Arrestin Recruitment Determined by Bioluminescence Resonance Energy Transfer (BRET)

BRET was determined essentially as described previously [34]. Briefly, HEK293 cells were transiently transfected with CCR7-EYFP or -HA and β-arrestin2-hRLuc at a ratio of 3:1 and were grown for 24 h. Cells were washed with PBS and re-suspended in PBS containing 5 mM glucose (PBS-G). The cell suspension was inoculated in a white 96F bottom half-well plate (Perkin Elmer, Waltham, MA, USA) in the presence of 10 µM coelenterazine H (Cayman Chemical, Ann Arbor, MI, USA) at 37 °C. Fluorescence and luminescence were recorded on a Spark 10M multimode reader (Tecan, Männedorf, Switzerland). Cells were stimulated after 9 min of recording with 50 nM of chemokines. EYFP fluorescence (505–590 nm, 350 ms) and luciferase bioluminescence (430–485 nm, 350 ms) were recorded over several subsequent cycles. BRET was calculated by dividing the EYFP emission by the luciferase emission, NetBRET by subtracting the BRET of EYFP-transfected wells from the BRET of HA-transfected wells, and ΔNetBRET by subtracting the NetBRET signal from the basal NetBRET.

### 4.8. Confocal Live-Cell Imaging

Live-cell confocal imaging was performed on a TCS SP5 II microscope (Leica, Wetzlar, Germany) equipped with a temperature-controlled chamber. The videos were routinely recorded with a 63× objective. Live cell imaging was conducted in 35 mm Mattek No. 1.5 dishes in a medium supplemented with 20 mM HEPES buffer pH 7.5.

## Supplementary Materials

Supplementary materials can be found at http://www.mdpi.com/1422-0067/19/12/3876/s1.

## Abbreviations

ACKR4	Atypical chemokine receptor 4
CCL19	CC chemokine ligand 19
CCL21	CC chemokine ligand 21
CCR7	CC chemokine receptor 7
DCs	Dendritic cells
ERK	Extracellular signaling regulated kinase
GAGs	Glycosaminoglycans
GPCR	G-protein coupled receptors
MoDCs	Monocyte-derived DCs
mRFP	Monomeric red fluorescent protein
PTx	Pertussis toxin
